# Green-synthesized natural antioxidants in poultry and livestock feed: enhancing oxidative stability and animal welfare

**DOI:** 10.3389/fvets.2025.1720052

**Published:** 2026-01-23

**Authors:** Khaoula Nefzi, Ikram BenSouf, Mariem Saidani, Cyrine Darej, Bochra Bejaoui, Manel Ben Larbi, Naceur M’Hamdi, Vincent Lequart, Nicolas Joly, Patrick Martin

**Affiliations:** 1 Laboratory of Management and Valorization of Forest Resources LR11INRGREF0, National Research Institute of Rural Engineering, Water and Forests (INRGREF), University of Carthage, Ariana, Tunisia; 2 Laboratory of Animal, Genetic and Feed Resources (LRGAA), National Agronomic Institute of Tunisia, University of Carthage, Tunis, Tunisia; 3 Higher School of Agriculture, University of Carthage, Mateur, Tunisia; 4 Laboratory of Research on Ecosystems and Aquatic Resources, National Agronomic Institute of Tunisia, University of Carthage, Tunis, Tunisia; 5 Laboratory of Useful Materials, National Institute of Research and Physico-Chemical Analysis (INRAP), University of Carthage, Ariana, Tunisia; 6 Faculty of Sciences of Bizerte, Department of Chemistry, University of Carthage, Bizerte, Tunisia; 7 Unité Transformations and Agroressources, ULR7519, Université d’Artois-Uni LaSalle, Bethune, France

**Keywords:** antioxidants, natural sources, feed additives, mechanism of action, physicochemical properties

## Abstract

Oxidative stress, caused by an imbalance between reactive oxygen species and antioxidant defenses, significantly impacts livestock health, welfare, and productivity. Green synthesis has emerged as a sustainable approach for enhancing the stability and bioavailability of natural antioxidants in animal feed. Unlike conventional extraction methods, green-synthesized antioxidants derived from plant extracts, essential oils, and agro-industrial by-products offer improved oxidative stability, reduced toxicity, and enhanced bioactivity. These bioengineered antioxidants not only mitigate oxidative stress but also support immune function, improve feed efficiency, and enhance meat quality by reducing lipid peroxidation and increasing vitamin E content. Furthermore, the incorporation of green-synthesized antioxidants in livestock nutrition contributes to environmentally friendly production practices, aligning with sustainable agriculture and consumer demand for natural animal products. This review examines the potential of green-synthesized antioxidants, their role in improving oxidative stability, and their impact on animal welfare, performance, and product quality.

## Introduction

Over the past two decades, interest in natural plant feed additives (PFA) as alternatives to synthetic antioxidants in livestock and poultry nutrition has grown significantly (1). Extensive research has explored the antioxidative potential of various bioactive compounds, leading to the development of novel antioxidant formulations and their incorporation into animal feed (2). Antioxidants play a crucial role in neutralizing free radicals and reactive oxygen species (ROS), thereby protecting cellular integrity and improving overall health (3). Their beneficial properties have been widely recognized in both food preservation (4, 5) and animal health applications (6–9).

The inclusion of natural antioxidants in animal feed has demonstrated the potential to enhance livestock performance, immune function, and oxidative stability, making them valuable in modern poultry and livestock farming (10). While fruits and vegetables are primary sources of natural antioxidants, Asif (11) identified additional sources from medicinal plants and agricultural by-products, broadening the scope of sustainable antioxidant applications. These plant-derived antioxidants, including polyphenols (phenolic acids, flavonoids, anthocyanins, lignans, and stilbenes), carotenoids (xanthophylls and carotenes), and essential vitamins (E and C), play a critical role in maintaining animal health and improving product quality (2).

With growing concerns over environmental sustainability and feed efficiency, green synthesis has emerged as a promising approach for extracting and delivering natural antioxidants. This method utilizes eco-friendly techniques to enhance antioxidant bioavailability, reduce feed spoilage, and improve oxidative stability while minimizing chemical solvent use. Currently, feed additives containing antioxidant compounds are authorized based on their efficacy in preventing lipid peroxidation, thereby prolonging feed shelf life. However, recent efforts have focused on sustainable extraction methods, activity assessment, and the identification of optimal dietary sources to improve feed efficiency and animal welfare (12).

**Figure 1:**
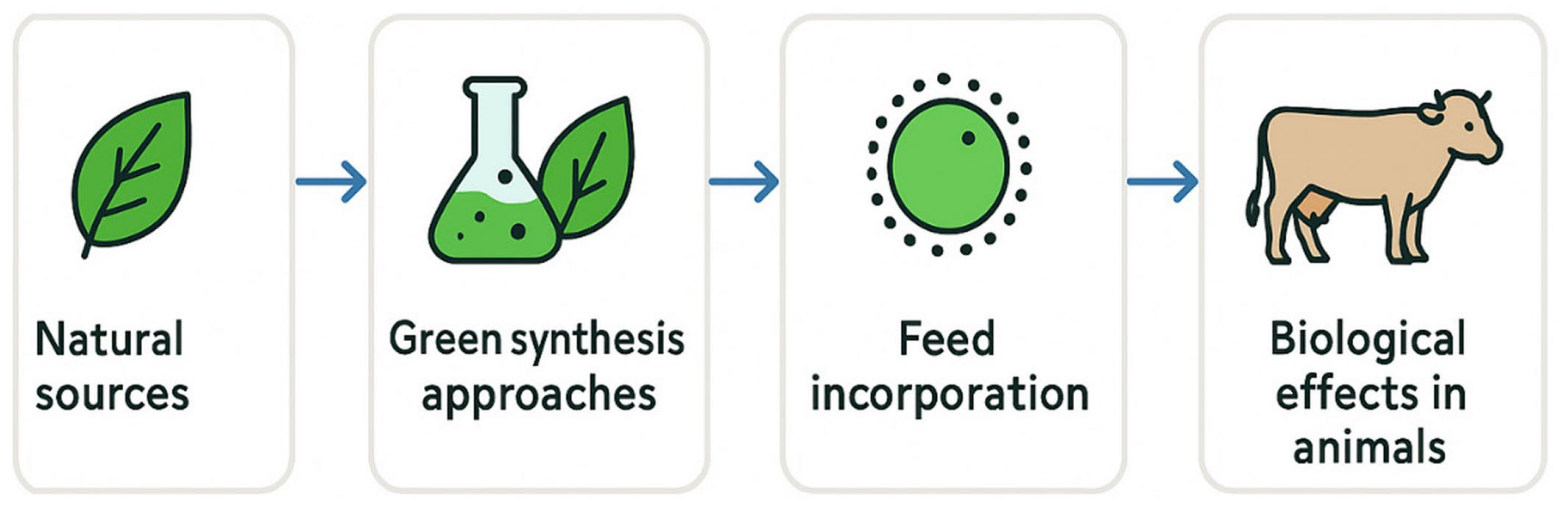
Conceptual workflow of green-synthesized antioxidants in animal nutrition.

This review provides a comprehensive overview of green-synthesized natural antioxidants, exploring their sources, extraction techniques, regulatory aspects, and applications in poultry and livestock nutrition. It specifically examines how these antioxidants enhance oxidative stability, animal health, and product quality, contributing to more sustainable and efficient livestock production systems (Figure 1).

## The natural sources of antioxidants

### Marines’ resources of antioxidants

Diaz et al. (13) reported that 90% of the planet’s biomass is found in the oceans, with marine species accounting for about 50% of the world’s total biological diversity. This expansive diversity of organisms is known as a store of effective molecules that are created by marine life forms to bolster their survival in a threatening environment (14). Among marine organisms, seaweeds have been identified as an under-exploited plant resource (15, 16). Since the 1940s, the generation of algal polysaccharides has come to commercial unmistakable quality through their application as a thickening and gelling agent for several industrial applications (17). Furthermore, it is well known that marine algae are abundant sources of biologically active chemicals with a wide range of chemical structures and promising pharmacological and therapeutic applications. According to research, chemicals derived from marine algae demonstrate a range of biological activity, including anti-coagulant (18), antiviral (19), antioxidant (20), and anticancer (21). In recent years, diverse sulfated polysaccharides (SPs) extracted from algae seaweeds have gained much attention in the food, pharmaceutical, and cosmetic industries. PSs include a complex group of macromolecules with a great range of interesting biological activities. These polymers are chemically anionic and are common in marine algae and animals, such as invertebrates (22). Seaweeds are the main source of non-animal PSs, and their chemical structures vary among species, such as carrageenan in red algae (Rhodophyceae), fucoidan in brown algae (Phaeophyceae), and ulvan in green algae (Chlorophyceae) (18). These PSs have displayed different biological activities beneficial to health such as anticoagulation (23), anti-HIV-1 (24), immunomodulatory (25), and anticancer (26).

Among the phenolic compounds, phlorotannins are composed of the polymerization of phloroglucinol, defined as 1,3,5-trihydroxy benzene, and biosynthesized by acetate-malonate. These are highly hydrophilic compounds with a wide range of molecular sizes ranging from 126 to 650.000 Da (27). Marine brown algae accumulate a diverse range of phloroglucinol-based polyphenols, such as phlorotannins, which could be employed as functional ingredients in nutraceuticals with potential health effects (28, 29). Among seaweeds, *Ecklonia cava*, an edible brown seaweed, is a more abundant source of phlorotannins than others (30). Phlorotannins have various biological activities beneficial to health, especially antioxidant (31), anti-HIV (32), antiproliferative (33), anti-inflammatory (34), radioprotective (35), antidiabetic (31), and antihypertensive (36). Carotenoids are pigmented compounds from plants, algae, fungi, and microorganisms. They are the main natural pigments responsible for photosynthetic organisms’ different colors (37). Nishida et al. (38) reported that carotenoids exhibit stronger singlet-oxygen quenching activity than *α*-tocopherol and α-lipoic acid, highlighting fucoxanthin from the brown algae *Undaria pinnatifida* and *Laminaria japonica* as particularly effective. Fucoxanthin, whether directly extracted from *U. pinnatifida* or obtained through lipase-mediated hydrolysis, also shows strong radical-scavenging capacity against DPPH and ABTS radicals (39). Furthermore, the cytoprotective action of fucoxanthin, from a brown alga Sargassum siliquastrum, against H_2_O_2_-induced cell destruction (39).

### Fruit and vegetable products

Due to their richness in various antioxidants, new and handled natural products (fruits and vegetables) are known for their powerful antioxidant activity. The content of different bioactive compounds in fruits and vegetables is related to the nature of the raw material. Products including fruit, vegetables, coffee, tea, herbs, and spices include polyphenolic substances such as flavonoids, phenolic acids, lignans, and stilbenes. Flavonoids include anthocyanins, which are found in berries, as well as flavonols (kaempferol, quercetin, and myricetin), flavanols (catechin, epicatechin), which are found in cocoa, dark chocolate, green tea, and black tea (40). Betalains, found in red beets, cactus pears, pitaya, and amaranth (41), and chlorophylls, prevalent in green leafy vegetables (42), are other substances with strong antioxidant capabilities. The consumption of berries has recently increased due to the high levels of polyphenols, which are known to have health benefits. Blueberries had greater levels of anthocyanins, flavonols, and phenolic acids, while Strawberries had higher levels of flavan-3-ols, dihydrochalcones, and flavanones. Anthocyanins were the most important phenolic constituents of both berries. Additionally, the higher total phenolic content of blueberry jam justified its higher antioxidant capacity as determined by the DPPH free radical assay, compared to strawberry. Among the different plants, natural products, and vegetables are known to supply health benefits (43–45). plant-derived natural products such as citrus fruits (oranges, grapefruit, lemons, and limes), grapes, pomegranates, apples, dates, green and yellow vegetables (peppers), cabbage, strawberries, carrots, green leafy vegetables, and bananas (46) are known globally to contain antioxidants. Antioxidants are recognized by their both added substance and synergistic activities in minimizing the hazard of chronic diseases (47). Hence, fruits and vegetables have protective functions against cardiovascular diseases. In general, the defending role of plant-derived natural products has been assigned to their antioxidant components (natural radical terminators) such as vitamins A, C, and E (*α*-tocopherol), *β*- and α-carotene, and glutathione (48). Other antioxidants such as alkaloids, terpenoids, sulfur compounds, and phenolic and polyphenolic compounds were found in plant-derived natural products (Table 1) (49), reducing oxidative damage by scavenging free radical activities (50). Moreover, these bioactive, non-nutritive plant compounds, for the most part, are assigned as phytochemicals, contribute to the end-of-chain responses by disposing of free radical intermediates (48). Niki and Noguchi (51) reported that carotenoids, an extremely important bioactive compound present in plant-derived natural products, are especially compelling in avoiding oxidation. Another group of bioactives, such as polyphenolic flavonoids, is plant metabolites with multiple organic and pharmacological properties (52, 53).

**Table 1:** Summary of the natural antioxidants and their sources.

Sources	Origin	Phytochemical class	Antioxidants	References
Marine sources	-Brown algae	-Polyphenols	- Phlorotannins	(31)
	- Undaria pinnatifida -Laminaria japonica -Sargassum siliquastrum	- Carotenoids	- Fucoxanthin	(39, 40)
	- Brown algae (Phaeophyceae) -Red algae (Rhodophyceae) -Green algae (Chlorophyceae)	Sulfated polysaccharides (SPs)	- Fucoidan - Carrageenan -Ulvan	(18)
Medicinal plants	*Cistus monspeliensis*	Phenolics acid	Caffeoyl shikimic acid, 3,4-dihydroxybenzoic acid-O-hexoside	(65)
		Flavonoids	Amentoflavone	
	Globularia alypum	Phenolics acid	Sinapic acid derivative	(65)
		Flovonoids	Myricetin, Kaempferol glucoside, Liquiritin, Amentoflavone	
	Aspilia Africana	Phenolics acid	Chlorogenic acid	(161)
		Vitamins	ascorbic acid, riboflavin, thiamine	
fruit	Apple	Sterols	Campesterol, β-sitosterol	(162–166)
		Anthocyanins	Cyanidin, delphinidin	
		Flavanols	Catechin	
		Flavanols	Quercetin, kaemferol	
		Dihydrochalcones	Phloretin	
		Hydroxycinnamic acids	Ferulic acid, chlorogenic acid	
		Salicylates		
	Berries	Hydroxybenzoic acids	Gallic acid	(54, 162)
		Flavanols	Catechin	
		Flavonols	Quercetin, kaempferol	
		Anthocyanins	Cyanidin, delphinidin	
		Stilbenoids	Resveratrol, pterostilbene, piceatannol	
	Banana	Hydroxybenzoic acids	Gallic acid	(54, 162–166)
		Flavanols	Catechin, epicatechin, epigallocatechin	
		Flavonols	Myricetin	
		Lignans	Pinoresinol	
		Sterols	Campesterol	
vegetable	Broccoli	Sterols	Campesterol, β-sitosterol	(162, 163, 167–169)
		Carotenoids	α-carotene, β-carotene, lycopene, xanthophylls	
		Quinones	Phylloquinone, menadione	
		Tocopherols and tocotrienols	α-tocopherol, β-tocopherol, *α*-T3, *β*-T3, α-tocotrienol, β- tocotrienols	
		Sterols	Sitosterol, β-sitosterol, sitostanol, campesterol, brassicaterol, stigmasterol, campestanol	
		Anthocyanins	Cyanidin,	
		Condensed tannins	Procyanidin A1, procyanidin B2	
		Glucosinolates	Progoitrin, sinigrin, glucoiberin, glucoraphanin, glucoalyssin, gluconasturtiin, gluconapin	
	Onion	Glycoalkaloids	α-solamargine, α-solasonine	(162)
		Sterols	Campesterol, β-sitosterol	
		Thiosulfinates	Allicin	
		Anthocyanins	Cyanidin, delphinidin	
		Flavonols	Quercetin, kaempferol	
	Spinach	Phenolic terpenes	Vitamin E	(162)
		Carotenoids	α-carotene, β-carotene, lycopene	
	Brussels sprouts	Carotenoid	β-carotene	(167–174)
		Tocopherols and tocotrienols	α-tocopherol, β-tocopherol, α-T3, β-T3, α-tocotrienol, β- tocotrienols	
		Glucosinolates	Progoitrin, sinigrin, glucoiberin, glucoraphanin, glucoalyssin, gluconapin, gluconasturtiin	
Agro-industry waste	Coffee	Anthocyanins	Delphinidin 3-*O*-(6′′-acetyl-glucoside), Peonidin 3-O-(6′′-acetyl-glucoside), Cyanidin 3-*O*-(6′′-malonyl-glucoside)	(63)
		Catechins	(+)-Catechin	
		Flavones	Apigenin	
		Hydroxybenzoic acids	Gallic acid 4-*O*-glucoside, Gallic acid 3-*O*-gallate, Gallic acid	
		Hydroxycinnamic acids	Caffeoyl aspartic acid, Caffeic acid 4-*O*-glucoside, Chlorogenic acid	
	Onion husks	Flavonols	Quercetin, 3′-Methoxy-4′,5,7-trihydroxyflavonol, Laricitrin	(175, 176)
		Flavanonols	Taxifolin	
		Flavonoid-*O*-glycosides	Quercetin-3,4′-*O*-di-β-glucoside, Isoquercetrin	
		Isoflavones	Tectorigenin	

### Medicinal plants

Natural products, particularly those based on plants, have been seen as important therapeutic alternatives (54) due to their richness in a wide variety of secondary metabolites with antimicrobial and antioxidant characteristics (55). Among these secondary metabolites, pharmacologically bioactive constituents are alkaloids, flavonoids, tannins, anthraquinones, and phenolic chemicals. *Cistus monspeliensis* and Globularia alypum are two Mediterranean-wide shrubs (56). The phytochemical examination illustrated that G. alypum and *C. monspeliensis* were rich in different compounds such as polyphenols, tannins, and flavonoids, which justifies their biological activities (57). In recent years, numerous researchers have conducted a comprehensive study on the qualitative structure of medicinal plant extracts. The chem profile of the genus Cistus was extremely variable due to geographical regions, subspecies variance, and soil-climatic conditions due to seasonal variations. The genus Cistus phenolic composition has been widely investigated and characterized by citing Cistus laurifolius, *Cistus incanus*, Cistus parviflorus, Cistus salvifolius, Cistus libanotis, and *Cistus creticus* (58). In the meantime, the extract of *C. monspeliensis* was found to contain numerous compounds from distinctive chemical classes such as flavonoids, coumarins, terpene derivatives, and hydrocarbons. The main compounds identified were isorhamnetin-O-rutinoside, isorhamnetin hexoside deoxyhexoside, and chrysoberyl di-glucoside (59). Thus, the chromatograms of the ethanolic extract of G. alypum disclosed a wide range of compounds; the most relevant are isorhamnetin-O-rutinoside, naringenin glucoside, tetragalloyl hexosid, myricetin, and I3, II8-Biapigenin (59). The medicinal plant Aspilia africana is owned by the Asteraceae family, and its leaves and roots have been exploited to treat many diseases such as wounds, osteoporosis, sores, malaria cough, febrile headache, wounds, gonorrhea, ear infections, stomachache, rheumatic pain, tuberculosis, measles, diabetes, diarrhea, gastric ulcers, and inflammatory conditions (60–62). The polyphenolic chemical class is primarily responsible for its antioxidant, anti-inflammatory, wound-healing, anticancer, antidiabetic, and antiulcer actions (60, 61).

### Agro-industry waste

Energy recovery and valorization of waste have become increasingly important in the context of environmental sustainability. Agro-industrial residues, in particular, are now widely recognized as promising feedstocks for biorefinery processes, where diverse biomasses can be transformed into value-added products. Through these conversion pathways, agro-industrial by-products can yield fuels, chemicals, energy, electricity, and a variety of functional compounds (63). Coffee-derived residues are a well-known example. Coffee pulp and parchment are naturally rich in polyphenols with strong antioxidant activity, which explains their growing use across sectors such as food and cosmetics. a wider and more representative range of residues, specifically grape pomace (rich in tannins, anthocyanins, and stilbenes), olive mill wastewater and olive pomace (hydroxytyrosol and oleuropein), tomato peels and seeds (lycopene and carotenoids), pomegranate peels (punicalagin and ellagic acid), date seeds (phenolics and dietary fibers), sugarcane bagasse (phenolic acids and flavonoids), as well as brewery spent grains and yeast biomass were used (64). However, because these by-products may also contain undesirable compounds, their industrial handling and processing remain challenging (65). Despite this, numerous studies have demonstrated their potential as raw materials for extracting antioxidant molecules for cosmetic applications (64, 66), for developing new composite materials (67), and even for water bioremediation (68, 69). Importantly, phenolic acids and other antioxidant constituents recovered from coffee waste can also be reintroduced into the coffee production chain as value-added food additives, further enhancing sustainability (70). The onion (*Allium cepa L.*) represents another major agro-industrial crop with significant potential for valorization. Consumed worldwide in raw and processed forms—including baking, boiling, braising, grilling, or frying (71)—global onion production has risen by approximately 25% in recent years (72, 73). This growth reflects both their culinary value and their richness in bioactive phytonutrients (72), many of which display antioxidant properties that help protect against oxidative stress (64, 71, 74). Epidemiological studies further suggest that regular onion consumption may reduce the risk of various cancers, as well as cardiovascular and neurodegenerative diseases (71, 74). With increasing production, onion processing generates substantial quantities of waste, a trend reflected in the surge of scientific interest reported in recent literature. Onion residues include skins, bark, husk, roots, bulb tops, and degraded bulbs (73, 75, 76), with outer skins alone accounting for up to 60% of total waste (73). These skins are particularly rich in polyphenols, notably quercetin and its glucosides—key flavonoids known for their strong antioxidant capacity. They also contain other valuable compounds such as ferulic acid, gallic acid, and kaempferol, which possess diverse biological activities (77).

### Extraction techniques of antioxidants

Extraction is a critical step in the investigation of natural antioxidants. Extraction processes represent an important step in producing antioxidants from food and medicinal plants (78, 79). According to Awad et al. (80), the extraction conditions and the processing protocols, such as solvent, time, temperature, and plant powder, should be optimized to obtain the optimum yield with the maximum concentration of active ingredients. Various extraction procedures, including green non-conventional methods, have been developed to improve the efficiency of antioxidant components extraction from plant materials (81, 82). Among the conventional extraction methods, aqueous extraction consists of extracting volatile organic and non-organic compounds with distilled water. This technique involves three processes: hydro-diffusion, hydrolysis, and decomposition by heat, and does not involve organic solvents (83). It can be used in combination with non-conventional technologies to increase the yield of volatile compounds (84). However, maceration in solvents and Soxhlet extraction are simple, low-cost, and fast (85). Still, they take a long time and require a lot of organic solvents, which have poor extraction yields (86, 87), and ultrasound extraction or modern methods such as supercritical and subcritical extraction and pressurized liquid extraction (88–90). Unconventional and environmentally friendly methods (ultrasonic, microwave (91), and pressure extractions (92)) have been developed to replace conventional methods. They have been connected alone or in conjunction with the use of solvents to decrease energy and solvent requirements (93).

It is alluring to have a better yield together with a noteworthy concentration of active compounds. Pressurized liquid extraction, supercritical fluid extraction, high hydrostatic pressure extraction, pulsed electric field extraction, and high-voltage electrical discharge extraction are new efficient ultrasound-assisted extraction techniques developed to increase extraction yields and decrease energy consumption (93). Microwave-assisted extraction of polyphenols was performed by Dahmoune et al. (94). EMA has been shown to have several advantages over conventional extraction methods, including higher extraction yield, lower solvent consumption, and shorter extraction time (95). These modern techniques are very effective and can be categorized as “green extraction” techniques (96, 97).

### Green synthesis: a sustainable approach for antioxidant delivery in livestock feed

Green synthesis offers a sustainable route for producing antioxidant compounds and delivery systems by relying on natural biological processes rather than harsh chemicals. It has gained attention as an eco-friendly method for generating bioactive materials, including natural antioxidants, through the use of plant extracts, microbes, and enzymes as reducing and stabilizing agents (98). This strategy avoids hazardous solvents and energy-intensive steps commonly found in conventional extraction and synthesis methods.

In this context, green-synthesized nanoparticles are nano-sized particles produced through these biological reactions. When plants rich in polyphenols, flavonoids, and other metabolites are used, their natural compounds act simultaneously as reducers (converting metal ions into nanoparticles) and stabilizers (preventing particle aggregation). Similar reactions can be driven by microorganisms or enzyme systems, resulting in nanoparticles with antioxidant properties or with the capacity to carry antioxidant molecules.

These biologically engineered nanoparticles enhance the stability and bioavailability of antioxidants by protecting them against degradation and improving their delivery within the digestive tract, thereby strengthening their capacity to reduce oxidative stress in livestock feed (99, 100).

A major contribution of green synthesis to livestock nutrition is its role in producing nanoparticles used for the nanoencapsulation of natural antioxidants. This approach enhances antioxidant stability and improves their bioavailability by protecting them from degradation and allowing a more controlled release throughout the digestive tract. Green-synthesized nanoparticles, such as silver, gold, or lipid-based carriers, have been shown to support oxidative stability in feed through these improved delivery characteristics (101). Beyond their functional advantages, green synthesis methods offer an important environmental benefit by reducing energy requirements and minimizing the generation of toxic byproducts during nanoparticle production (102). Incorporating green-synthesized antioxidants into poultry and livestock feed has been shown to strengthen immune function, reduce oxidative stress–related disorders, and ultimately promote better overall animal welfare (103). These benefits align with the growing shift toward sustainable livestock production and the development of eco-friendly feed additives.

A key component of this emerging field is Green Nanotechnology, which applies environmentally friendly synthesis techniques to produce nanoparticle-based delivery systems for antioxidants. Green-synthesized nanoparticles, such as silver, gold, and lipid-based particles, play an important role in the nanoencapsulation of natural antioxidants. By encapsulating these compounds, the nanoparticles help protect them from oxidation and degradation during feed manufacturing and digestion, ensuring that the antioxidants reach the intestinal tract in a more stable and active form (104, 105). This controlled-release capacity enhances absorption and bioactivity, ultimately improving the efficiency of antioxidant supplementation in livestock and poultry systems.

What distinguishes these technologies from traditional extraction and formulation methods is their reduced environmental footprint. Conventional approaches often require chemical solvents and high energy inputs, whereas green synthesis relies on biological agents, mild reaction conditions, and minimal toxic byproducts. As a result, green nanotechnology provides a cleaner and more sustainable pathway for producing antioxidant-rich feed additives (106, 107). This positions it as a promising strategy for large-scale implementation in animal nutrition while supporting broader goals of sustainable agriculture and environmental stewardship.

### Antioxidant properties

An antioxidant could be a substance able to prevent the oxidation of other molecules (108). The natural antioxidants are principally polyphenols (phenolic acids, flavonoids, anthocyanins, lignans, and stilbenes), carotenoids (xanthophylls and carotenes), and vitamins (vitamin E and C) (109, 110). Phenolic compounds present a diversified structure, ranging from simple molecules (ferulic acid, vanillin, gallic acid, and caffeic acid) to polyphenols (tannins and flavonoids) (111, 112). The most important Vitamins are vitamins E and C. Vitamin C is fat-soluble and composed of a group of chemical compounds consisting of four tocopherols and four tocotrienols, which include four isomers (*α*, *β*, *γ*, and *δ*) (113). In choosing natural plant extracts for human diets, the organoleptic qualities of the food product are considered (114). Many studies have shown that antioxidants have LD50 values lower than 1,000 mg/kg body weight, and ought not to have any critical impacts on animal performance (115, 116).

Natural antioxidants respond with free radicals or precursor metabolites, changing them into less reactive molecules and anticipating or postponing the oxidation of natural molecules. The most important and well-characterized natural antioxidants in the animal body are vitamins E and C. When the antioxidant system finds itself in high-stress conditions, if free radical production is increased dramatically, then without external help, it will be difficult to prevent damage to organs and cells. Increased dietary supplementation with natural antioxidants, particularly minerals like selenium, can offer this external assistance. Given that antioxidants are often expensive dietary components, it can be difficult for nutritionists or feed formulators to determine whether the antioxidant team in an animal body needs assistance and how much of this assistance might justify additional feed costs. The following are a few examples of potential pressures in the production of poultry (117).

Antioxidants shield physiologically significant molecules, such as DNA, proteins, and lipids, against deterioration (118). Supplementing with more antioxidants to increase meat quality while it’s being stored (119). Combining vitamin E and selenium can significantly minimize drip loss (120). Due to reduced vitamin E in the diet, the drop in egg production brought on by heat-related stress is increasing. Boosting antioxidant intake lowers mycotoxin toxicity and provides strong support for the body’s immune system (121).

The study’s findings led to the conclusion that antioxidants (vitamins E and S) may be combined with a baseline diet to get the greatest outcomes in terms of body weight increase. The superior performance may be attributable to the vitamins’ combined synergistic effects on the birds’ physiological systems (122). Numerous studies have demonstrated that natural antioxidants contain antioxidant, anti-inflammatory, metabolism- and immunity-modulating properties, as well as anthelminthic, anti-methanogenic, and antibacterial actions that are particularly significant in the production of cattle. These traits encourage research and education on these secondary metabolites’ potential applications as organic tools to improve animal performance and the quality of animal products (123).

### Use of antioxidants in livestock production and their effect on animal health, performance, and product quality

Animals are frequently subjected to a variety of oxidative stress circumstances that can influence animal health, decrease growth performance and production, and ultimately damage economic profitability. The addition of antioxidants to animal diets would be an important nutritional strategy to mitigate the negative effects induced by oxidative stress conditions (124). The addition of antioxidants as nutritional supplements in animal diets is a common practice to improve animal performance, health, and welfare (125). The use of antibiotics in animal production affects human and animal health, as well as the safety of animal products (126). Phytogenic feed additives have been used as alternatives to antibiotics for their potential effects in enhancing growth performance and quality characteristics of the derived products, including meat, milk, and eggs (127). During oxidative stress, unfavorable substances, including malondialdehyde (MDA), lipid peroxides (LPOs), and carbonyl protein complexes, could be formed and consequently cause organism damage and meat quality deterioration (128). Thus, feeding an animal with exogenous antioxidants provides oxidative stability, sensory quality, and the acceptability of derived products (129). Recently, numerous studies showed that polyphenol compounds, due to their contents of secondary metabolites, could maintain an antioxidant capacity as an important factor in animal health and exert their favorable effects in improving performance (128, 129).

### Ruminants

To prevent oxidative food deterioration, antioxidants have been widely employed as feed additives for cattle, sheep, and goats. Khalil et al. (130) explored the potential of orally administered moringa oil (MO) or its microencapsulated form (MON) to protect ram spermatozoa during cryopreservation by assessing their effects on semen quality, antioxidant capacity, apoptosis, seminal metabolic enzyme activity, as well as molecular docking study. A study by Alfaraj et al. (131) evaluated the potential of supplementing sheep diets with cobalt (CoNPs), iron (FeNPs) nanoparticles, or *Spirulina platensis* (SP) to tackle the adverse impacts of heat stress. They found that All nanozyme or SP treatments significantly (*p* < 0.05) enhanced growth performance, achieved the best results regarding hematocrit (*p* < 0.01) and platelets (p < 0.01), and exhibited lower WBC and lymphocyte counts, and higher globulin levels in comparison to stressed sheep. In bovine production, especially in herds that had managed contagious mastitis, vitamin E and selenium were associated with the prevalence of clinical mastitis and bulk tank Somatic cell count (SCC). Low bulk tank SCC and lower rates of clinical mastitis were linked to high serum Se levels. Up until cows ingested more than around 5 mg of selenium daily, the levels of selenium in blood and the feed were positively correlated (132). Se consumption had little effect on serum Se levels above this point. The percentage of clinical mastitis was adversely linked with the concentration of vitamin E in the diet. Vitamin E consumption was favorably correlated with plasma vitamin E concentrations; however, in dry cows as opposed to nursing cows, it had a stronger impact on serum vitamin E values (133). On the other hand, Malmuthuge and Guan (134) studied the effect of rumen protective glucose (RPG) supplementation on hepatic oxidative/antioxidant status and protein profile. In early postpartum cows, which may be at high risk for hepatic metabolic problems, many studies demonstrated that RPG decreased insulin sensitivity but raised triglyceride levels and oxidative stress. A study by Kong et al. (135) showed the importance of using the culture of Acremonium terricola (ATC) or ATC as a new feed additive in the diet of dairy cows. Indeed, ATC improved milk production and protein content. Kong et al. have suggested that this is strongly linked to an improvement in the immune system and the antioxidant capacity of ATC.

### Poultry

The internal content of antioxidants that slow down the oxidative effects in meat may be increased naturally by adding natural antioxidants to feed. The use of green-synthesized curcumin, chitosan, and silver nanoparticles has improved feed efficiency, intestinal morphology, immune response, and resistance to oxidative stress (128). Rosemary (*Rosmarinus officinalis L*.), which influences the further preservation of chicken meat and semi-finished products derived from it, is one of the sources of natural antioxidants for the poultry sector. *In vivo* tests revealed that grape seed extract prevents the oxidation of chicken lipids during stomach digestion (136). Antioxidants in liposomal form boosted the detoxifying capacity of laying hens and decreased the levels of xenobiotics, nitrites, and nitrates. The increased excretion of heavy metals from chicken bodies also avoided the buildup of residual heavy metals in the diet. The primary physiological and productivity markers of broiler chickens changed favorably when the liposomal nanoform of silymarin was added to their diet (137). Wang et al. (138) found that oxidative stress can decrease ovarian function, egg-laying performance, and affect body metabolites in the layered model. They then showed in their study the ameliorating effect of melatonin on ovarian oxidative stress, via the SIRT1-P53/FoxO1 pathway. Moreover, the beneficial effect of dietary supplementation with green-synthesized metal nanoparticles using plant extracts (AgNPs, AuNPs, ZnONPs) on the health, meat quality traits of chicken, productive traits (body weight, body weight gain, FCR), and antioxidant status of broiler chickens were demonstrated (138–142). Abbassi et al. (128) revealed the decrease of lipid oxidation in meat with the supplementation of a broiler diet with different sources of antioxidants (Vitamin E, rosemary, and thyme). Additionally, carnosine as an antioxidant can be efficiently utilized in chicken diets as a natural source of antioxidants and immunostimulants. Cong et al. (143) showed that carnosine supplementation in the animal diet improved meat quality, antioxidant activity, and decreased the lipid peroxidation status of breast meat.

### Pigs

Several natural antioxidants are available for use in the swine industry (144). Many studies showed that the incorporation of antioxidants, low vitamin E (Vit E as DL-*α*-tocopheryl acetate) levels, in the diet of pigs can reduce the negative effects of lipid peroxidation (145, 146). In swine, selenium nanoparticles produced through green synthesis have been used to enhance growth rate, strengthen immunity, and improve meat quality by reducing oxidative deterioration (145). Along the same lines, Lu et al. (147) found that the dietary addition of natural antioxidants was effective in improving growth. On the other hand, there is evidence in swine that antioxidants improve immune status (148, 149) and have potential health benefits for both animals and consumers (150). A study by Su et al. (151) reported that supplementing the diet of weaned pigs with antioxidants (i.e., natural antioxidant blend including polyphenols) increased body weight gain (BWG), serum IgG and IgA. Similarly, Malondialdehyde (MDA) levels decreased in serum, jejunal mucosa, and pancreas, while glutathione (GSH) levels significantly increased in serum, duodenal mucosa, and ileal mucosa.

### Horses

During stressful conditions in horses, including exercise, the body’s antioxidant levels must be adapted to cope with the ROS resulting from increased oxygen consumption (152). Horses competing in races are prone to antioxidant deficiencies (153, 154). Depending on the horse’s condition, it is necessary to supplement it with antioxidants (155). Antioxidant supplementation before stress (travel, competition, etc.) in horses is potentially beneficial to horses by enhancing immune function and protecting muscle and nerve cells (156). Miller et al. (157) showed that providing aged horses with an antioxidant supplement (Winergy Ventil–ate®, MARS Horsecare UK, Milton Keynes, UK), for 3 weeks before and after short-term transport helped reduce inflammation and modulate immune responses. Adah et al. (158) found that melatonin given before exercise reduced post-exercise biomarkers of oxidative stress (lower MDA and altered antioxidant enzyme activities) and improved some hematological parameters in Arabian stallions. Antioxidants have been shown to protect against equine protozoan myeloencephalitis (EPM), equine degenerative myeloencephalopathy (EDM), and fatigue during exercise in the equines (159, 160).

## Conclusion

In recent years, there has been increasing interest in integrating natural bioactive compounds as sustainable nutritional alternatives for livestock and poultry. Antioxidants play a crucial role in immune responses, cell signaling, transcription factor activities, and gene expression, contributing to overall animal health and welfare. The literature reviewed in this study highlights the significance of various plant-based antioxidants, considering their bioavailability, active compounds, and geographic accessibility. With increasing concerns over feed quality, oxidative stability, and consumer safety, green-synthesized antioxidants have emerged as eco-friendly alternatives to synthetic preservatives in livestock production. Unlike conventional antioxidants, green-synthesized compounds enhance lipid oxidation resistance while minimizing environmental impact and potential health risks. Recent research underscores their effectiveness in mitigating oxidative stress, improving feed efficiency, and supporting sustainable animal production. As the demand for natural, high-quality animal products continues to rise, integrating green synthesis techniques in antioxidant production presents a promising approach to enhance livestock welfare, performance, and oxidative stability. Future research should focus on optimizing green extraction methods, delivery systems, and regulatory frameworks to maximize the benefits of sustainable antioxidant applications in animal nutrition.
